# Supplementary material for: The Elevated Expression of ORF75, a Lytic KSHV Gene, in Kaposi Sarcoma Lesions is Driven by a GC-rich DNA cis Element in its Promoter Region
Source: bioRxiv. 2024 Sep 26:2024.09.26.615194. Preprint. [Version 1] doi: 10.1101/2024.09.26.615194 (PMC12478400; doi:10.1101/2024.09.26.615194)
Supplement: Supplement 1 [file NIHPP2024.09.26.615194v1-supplement-1.pdf]

(which was not certified by peer review) is the author/funder. This article is a US Government work. It is not subject to copyright under 17 U
105 and is also made available for use under a CC0 license.

A)

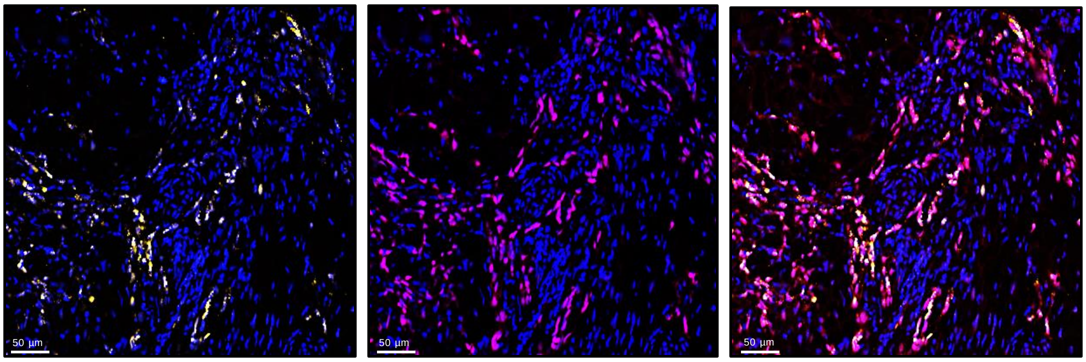

Figure S1: Dual stained zoomed-in KS skin tissue region. A) KS Skin lesion zoomed in section from Figure A, showing colocalization of ORF75 RNA and LANA protein.

(which was not certified by peer review) is the author/funder. This article is a US Government work. It is not subject to copyright under 17 U
105 and is also made available for use under a CC0 license.

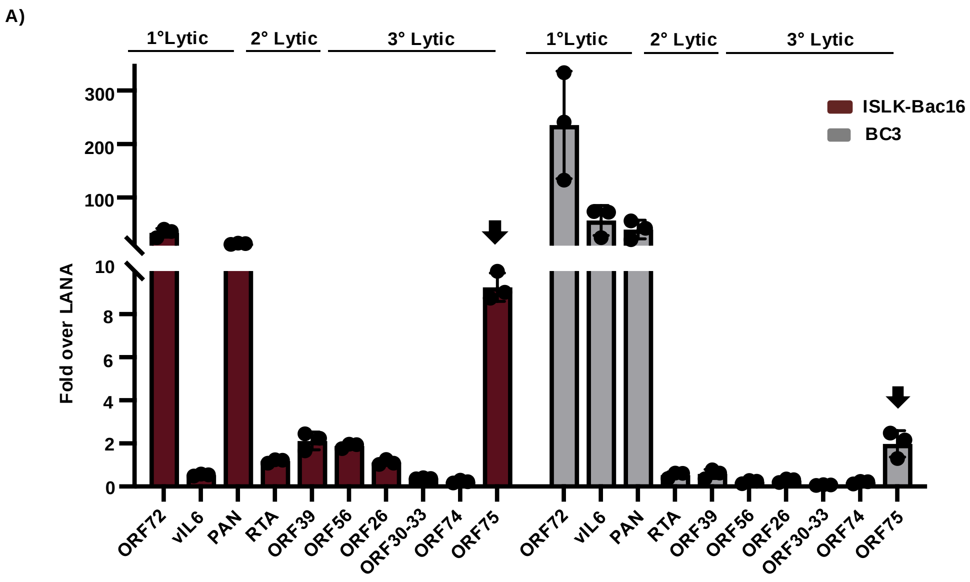

Figure S2: KSHV gene expression analysis in a infected epithelial and a B-cell line. A) qPCR analysis of representative genes of latent and lytic cycle in latently infected iSLK-BAC16 and PEL cell line, BC3. N=3 biological replicate with three qPCR technical replicate. Passage 6, 9 and 14 for ISLK-BAC16 cell line. Expression normalized to uninfected iSLK cells and uninfected BJAB cells for infected iSLK-BAC16 cells and BC3 cells ,respectively with GAPDH as internal reference. Expression of all genes were fold normalized to respective LANA expression for each cell type. Shown are the means ± standard deviations of at least 3 separate experiments.

(which was not certified by peer review) is the author/funder. This article is a US Government work. It is not subject to copyright under 17 U
105 and is also made available for use under a CC0 license.

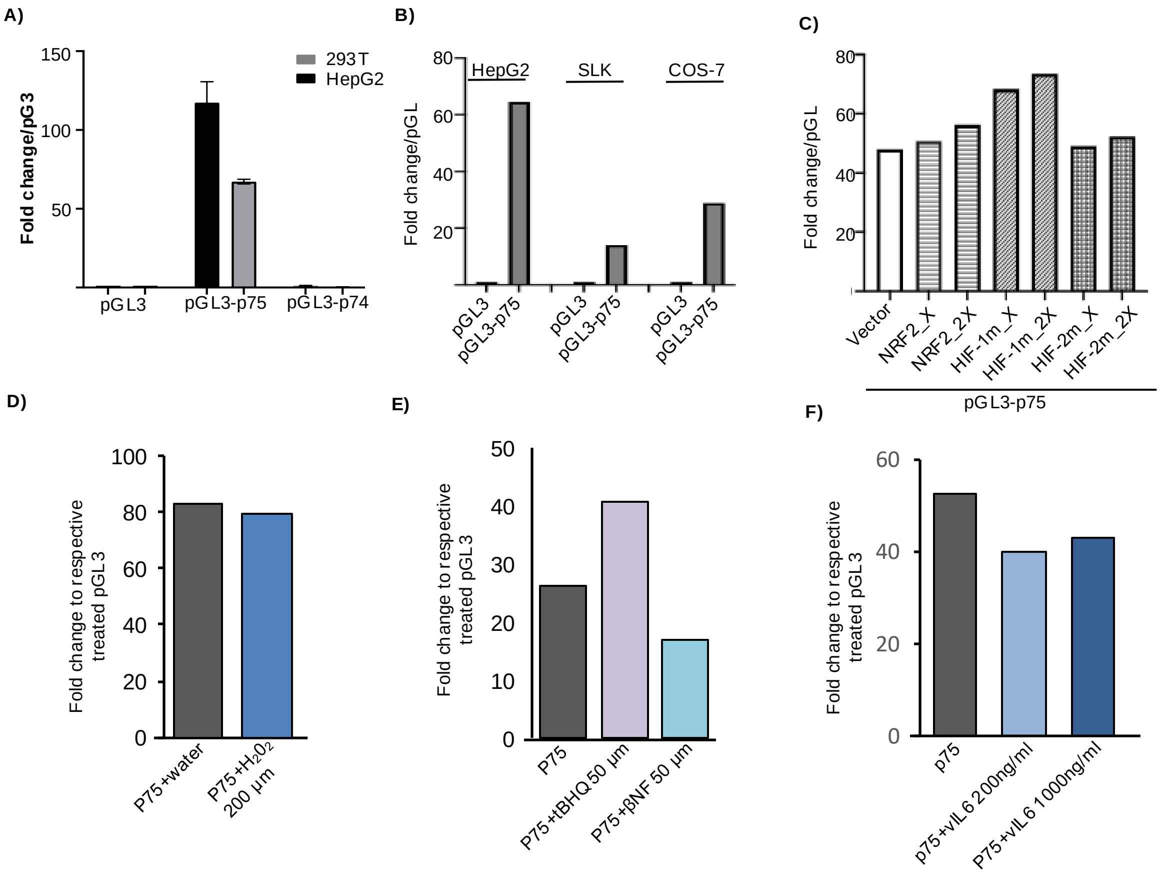

Figure S3: Basal ORF75 promoter activity is independent of HRE and ARE elements. A) Promoter luciferase assay of ORF75 and ORF74 (1.2 kb) promoter in HEK293T and HepG2. Data normalised to respective pGL3 vector in each cell line. Histogram represents mean with SD as error bars for three biological replicate. Assayed at 72h post transfection. **B)** Promoter luciferase assay of ORF75 promoter in HepG2, SLK and COS-7 cells. Assayed at 72h post transfection. **C)** Promoter luciferase assay of the ORF75 promoter in HepG2 cells, coupled with transient overexpression of NRF2, HIF1α, and HIF-2α degradation-resistant mutants. X and 2X indicate 1:2 and 1:4 ratios of ORF75 promoter to protein expression plasmid, respectively. **D, E, F)** Promoter luciferase assay of ORF75 promoter in HepG2 cells with various treatments. All treatments were done 24h post transfection. Assayed at 48h. Histogram for all except A, one experiment with two technical replicates. Error bar indicate ±SD.

(which was not certified by peer review) is the author/funder. This article is a US Government work. It is not subject to copyright under 17 U
105 and is also made available for use under a CC0 license.

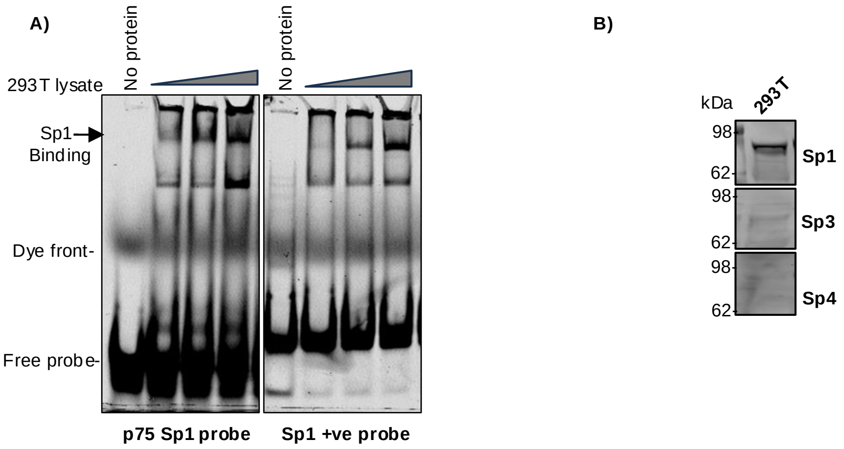

Figure S4: ORF75 promoter's proximal Sp1 element is regulated by Sp1 transcription factor. A) EMSA showing binding of Sp. proteins with dsDNA probe of ORF75 promoter along with a positive control Sp1 probe (Li-COR, P/N: 829-07926) in a 8% native PAGE gel. **B)** WB: HEK293T whole cell lysates was probed for Sp1, Sp3 and Sp4 protein abundance. See Figure S7 for full blots.

(which was not certified by peer review) is the author/funder. This article is a US Government work. It is not subject to copyright under 17 U
105 and is also made available for use under a CC0 license.

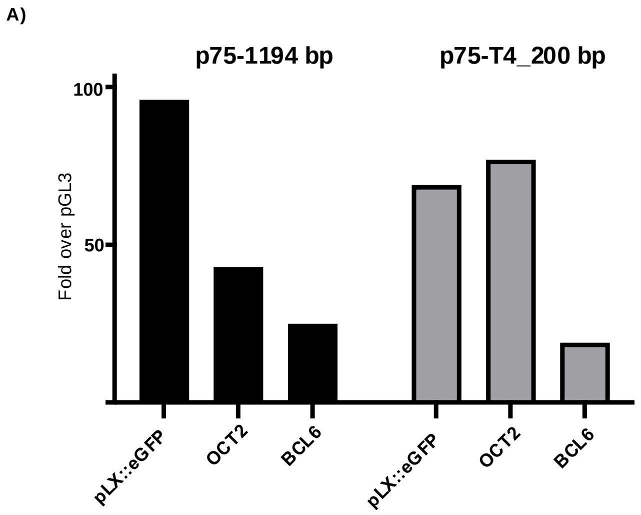

Figure S5: Promoter repressor assay. A) Promoter luciferase assay of full length ORF75 (p75) and truncated promoter construct (p75-T4) with co-expression of OCT2 and BCL6 expression constructs. eGFP expression plasmid was used as vector control.

(which was not certified by peer review) is the author/funder. This article is a US Government work. It is not subject to copyright under 17 U
105 and is also made available for use under a CC0 license.

A)

Name	Minimum	Maximum	Adjacent gene
SP1	137878	137887	TR
SP1	134601	134610	ORF75
SP1	119251	119260	Repeat region
SP1	85831	85840	vIRF-1
SP1	81510	81519	ORF57,56
SP1	71583	71592	ORF45-50
SP1	59739	59748	ORF39
SP1	51876	51885	ORF29-33
SP1	24014	24023	Repeat region
SP1	18270	18279	ORF2, vIL6 (K2)

B)

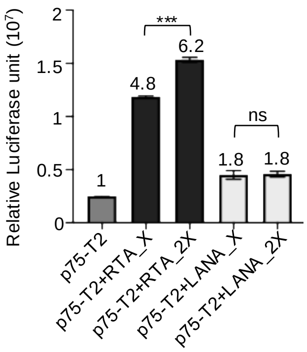

D)

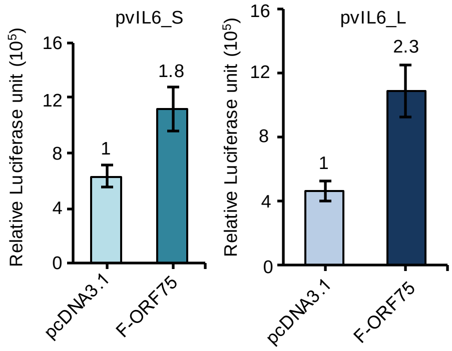

C)

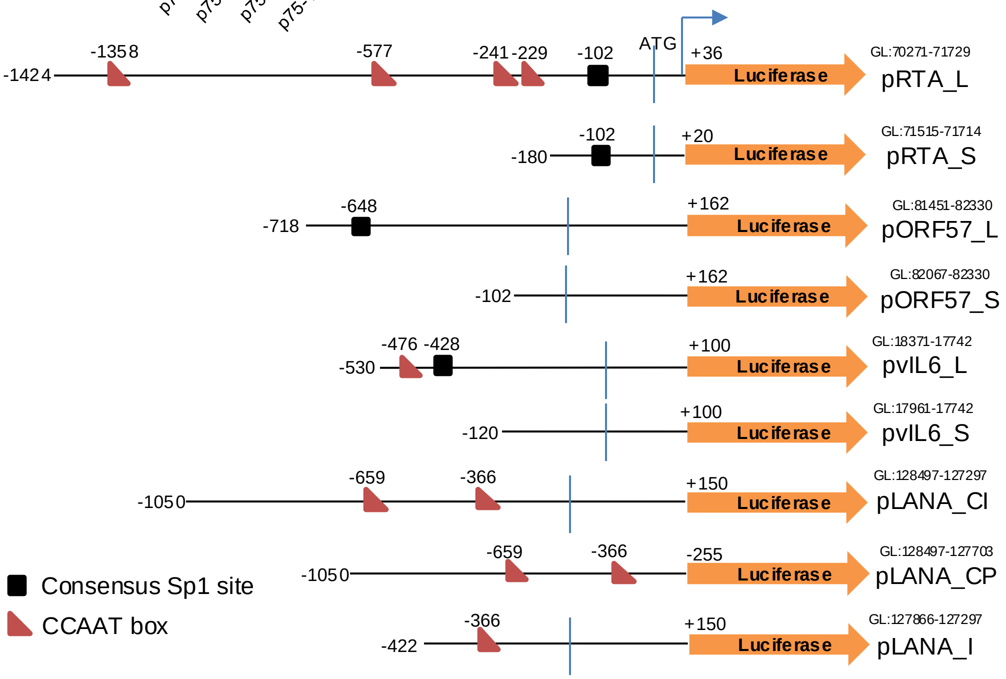

Figure S6: Multiple consensus Sp1 elements are present across KSHV genome. A) Table showing multiple consensus Sp1 element position throughout KSHV genome. Only complete consensus sequence is shown here. Consensus sequence KGGGCGGRRY, where K stands for G or T and R stands for G or A. **B)** Promoter luciferase assay of ORF75-T2 promoter in HEK293T cells along with co-expression of increasing amount of RTA and LANA protein. X and 2X indicate 1:2 and 1:4 ratios of ORF75 promoter to protein expression plasmid, respectively. Assayed at 72h post transfection. Shown are the means ± standard deviations of 3 separate experiments. *P*-values (***$p \leq 0.001$, ns not significant) are calculated using two-sided unpaired *t*-test. **C)** Schematic diagram of various KSHV gene promoters used in this study. **D)** Promoter luciferase assay of vIL6 promoters along with co-expression of F-ORF75. Error bar indicate ±SD, N=3. GL: genomic location as per NC_009333 KSHV reference genome.

(which was not certified by peer review) is the author/funder. This article is a US Government work. It is not subject to copyright under 17 U
105 and is also made available for use under a CC0 license.

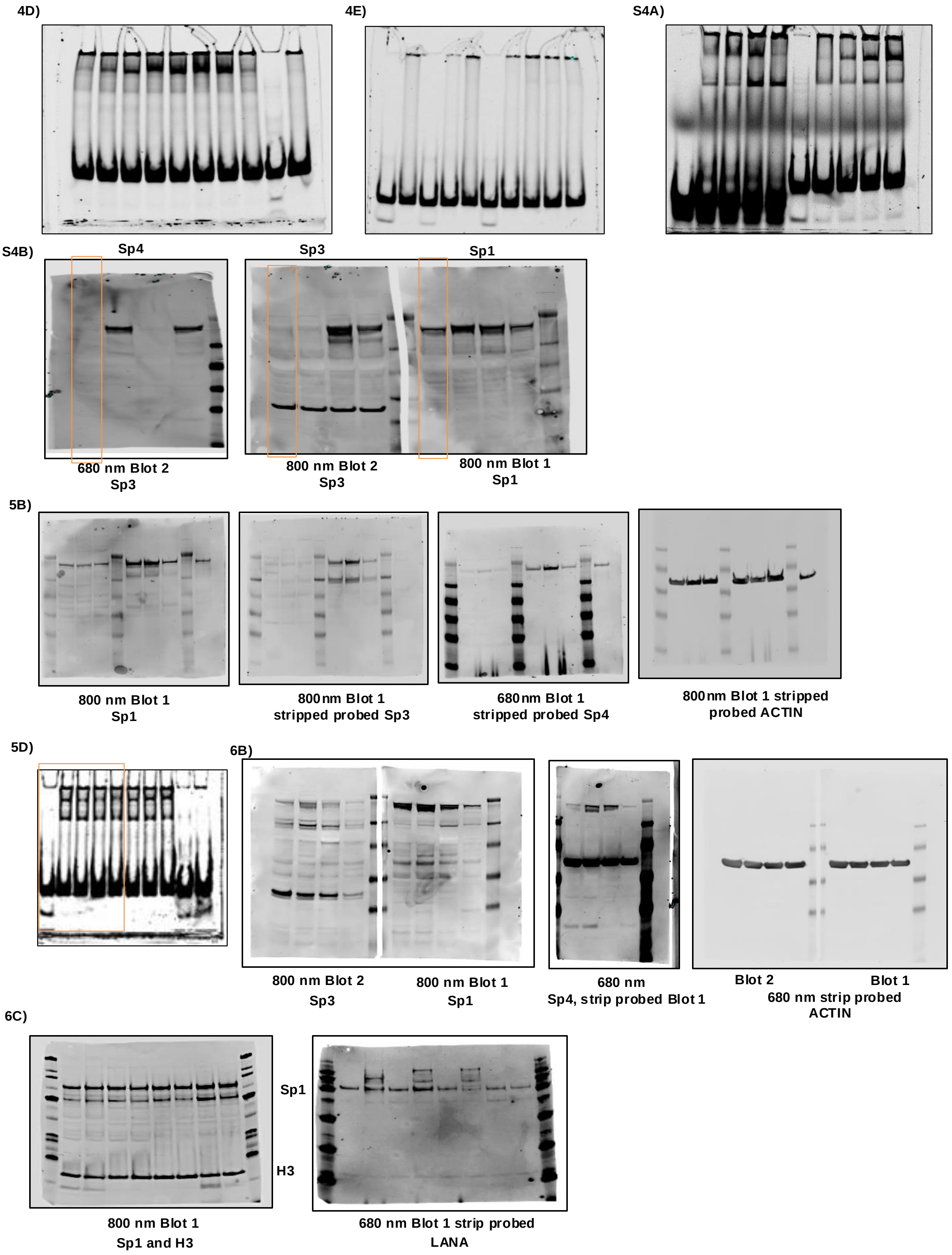

Figure S7: Complete, uncropped EMSA and western blots. Figure labels used for all uncropped blots here are same as the figure labels used in the cropped blots. 680 and 800 nm indicates the *LICOR* IR dye channel used for scanning. Indicated blot number can be used to trace stripping and reprobing order.

(which was not certified by peer review) is the author/funder. This article is a US Government work. It is not subject to copyright under 17 U
105 and is also made available for use under a CC0 license.

7A

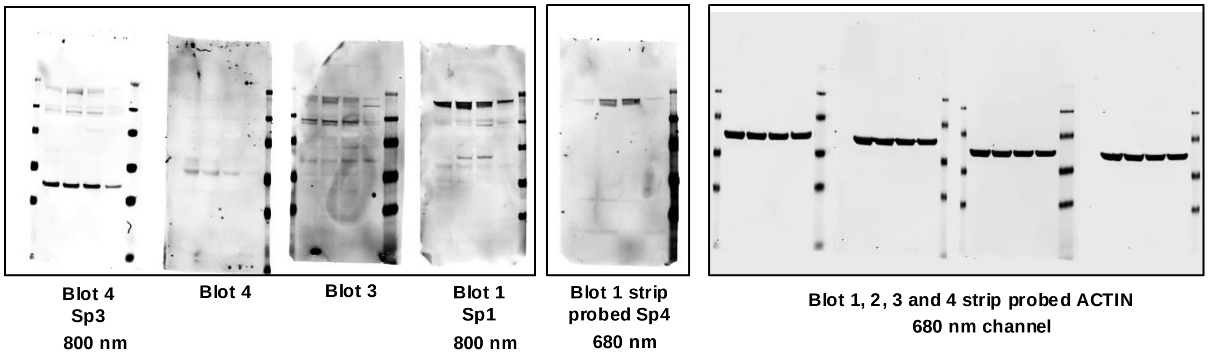

7B

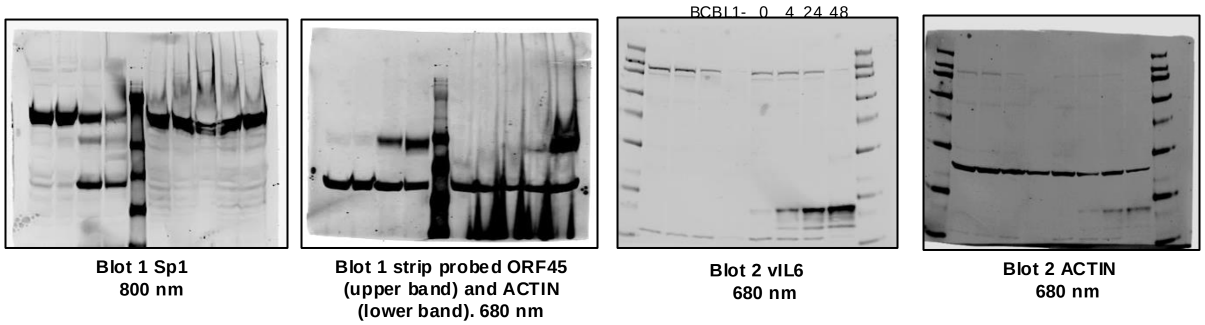

7C

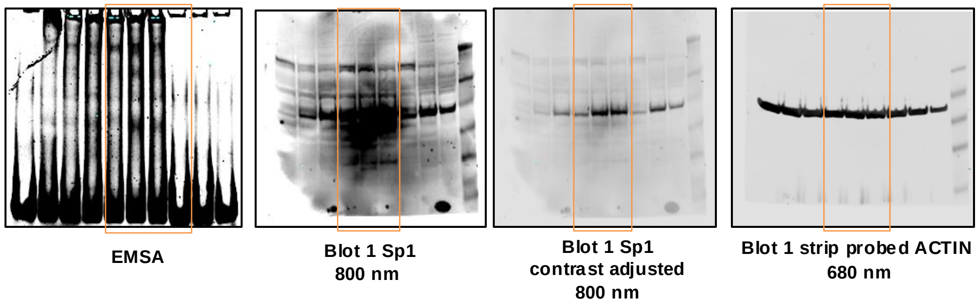

7D

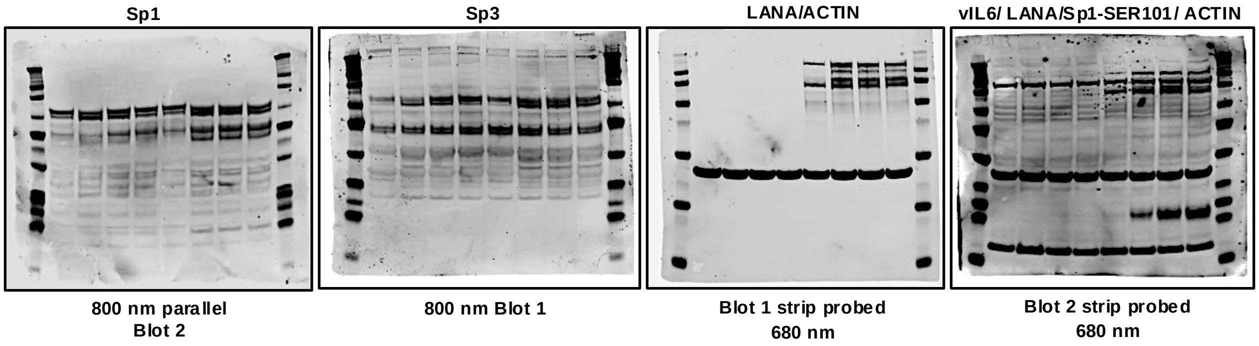

8A

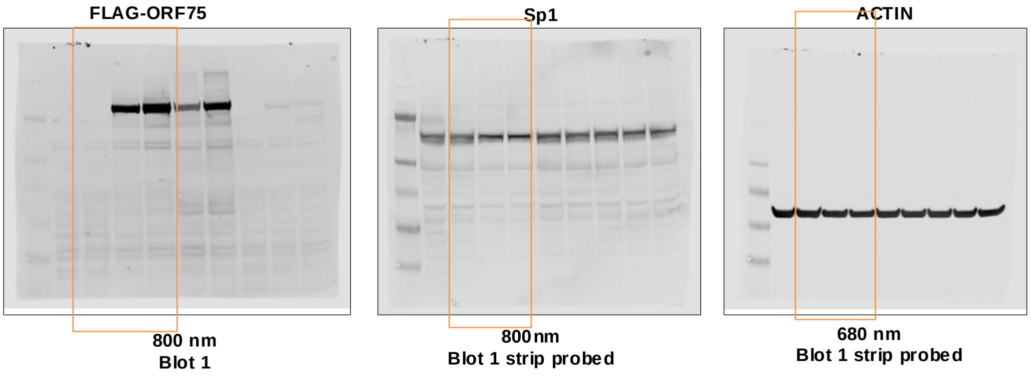

bioRxiv preprint doi: https://doi.org/10.1101/2024.09.26.615194; this version posted September 26, 2024. The copyright holder for this preprint (which was not certified by peer review) is the author/funder. This article is a US Government work. It is not subject to copyright under 17 USC 105 and is also made available for use under a CC0 license.

Table S1: List of primers used in the study

Gene	Primer sequence	Reference	Experiment
RTA	F: TTGCCAAGTTTGTACAACTGCT<br>R: ACCTTGCAAAGACCATTCAGAT	Wang et al. 2020	qPCR
vIL6	F: CTGTTACCGTACCGGCATCT<br>R: GGGTGGACTGTAGTGCGTCT	Wang et al. 2020	qPCR
Actin	F: CCTTCCTGGGCATGGAGT<br>R: CAGGGCAGTGATCTCCTTCT	Wang et al. 2020	qPCR
18S	F: GCCCGAAGCGTTTACTTTGA<br>R: TCCATTATTCCTAGCTGCGGTATC	Veeranna et al. 2012	qPCR
ORF72	F: CATTGCCCGCCTCTATTATCA<br>R: ATGACGTTGGCAGGAACCA	Vladimirova et al. 2023	qPCR
LANA	F: GTGACCTTGGCGATGACCTA<br>R: CAGGAGATGGAGAATGAGTA	Veeranna et al. 2012	qPCR
ORF74	F: CAAGCAGGCCATGTGTTATG<br>R: AGCACCACAGCAACAATCAC	This study	qPCR
PAN	F: CGGTGTTTTGGCTGGGTTT<br>R: AAACCTTGCCGTCTGGTCACT	Vladimirova et al. 2023	qPCR
ORF75 coding	F: TATACAAGCCGTACGGGCAC<br>R: CTCGAAGTGGGAGGTCTTCG	Vladimirova et al. 2023	qPCR
ORF56	F: GGTCCACAGATTCCCGTCAA<br>R: GGGGAGTGATGGAGCAGTTC	This study	qPCR
ORF45	F: CGTCCGGAGAGTTGGAACTG<br>R: GCGATCGTCGACCTGACAT	This study	qPCR
ORF39	F: TTTCCACCGAGTCAGCAGTG<br>R: ACACGTACTTATGGCAGCCT	This study	qPCR
ORF30	F: GCATTTCCTGCCCTGGTTCT<br>R: CAGCCTCTGCAGTTCCTCC	This study	qPCR
ORF75 endogenous	F: ATCACTCTCCAACCACAGCC<br>R: CACAGGTTGCTCTGCAGAGT	This study	qPCR
DHFR	F: TCGCCTGCACAAATAGGGAC<br>R: AGAACGCGCGGTCAAGTTT	Kaeser et al. 2002	ChIP-qPCR

bioRxiv preprint doi: https://doi.org/10.1101/2024.09.26.615194; this version posted September 26, 2024. The copyright holder for this preprint (which was not certified by peer review) is the author/funder. This article is a US Government work. It is not subject to copyright under 17 USC 105 and is also made available for use under a CC0 license.

P1-ORF75	F: ACTCCTGCAGTCCTAGCTCT<br>R: CACCGCTCCTTGGATATCCC	This study	ChIP-qPCR
P2-ORF75	F: GGGATATCCAAGGAGCGGTG<br>R: GCACACTCACTCTCGTACCC	This study	ChIP-qPCR
P3-ORF75	F: ATCACTCTCCAACCACAGCC<br>R: ACTCTGCAGAGCAACCTGTG	This study	ChIP-qPCR
P4-ORF75	F: CACAGGTTGCTCTGCAGAGT<br>R: CCTCCACGACCACAGACTTT	This study	ChIP-qPCR
P5-ORF75	F: AAAGTCTGTGGTCGTGGAGG<br>R: TCACCACAGCCAGACCAATC	This study	ChIP-qPCR
P6-ORF75	F: TCTGGCTGTGGTGATGGTTC<br>R: GGGAGCCAACTACTGAACGA	This study	ChIP-qPCR

Table S2: List of antibodies used in the study

Anti-ORF75	HAMB custom Rabbit polyclonal, Genscript Inc.
Anti-LANA	HAMB custom mouse monoclonal. Advanced Biotechnologies Inc., Columbia, MD
Anti-Sp1	Proteintech 21962-1-Ap, rabbit polyclonal.
Anti-Sp3	Proteintech 26584-1-Ap, rabbit polyclonal.
Anti-ORF45	Abcam, 2D4A5, Mouse monoclonal.
Anti-vIL6	HAMB custom mouse monoclonal
Anti-FLAG	Cell Signaling Technology, 14793
Anti-Sp4	Sc-390124 (B-1), Santa Cruz
